# Assessment of Informed Consent and the Impact of Simulation on Anesthesia Trainees

**DOI:** 10.7759/cureus.19787

**Published:** 2021-11-21

**Authors:** Muhammad Adeel Bashir, Asma A Khan, Sanaa a Khan

**Affiliations:** 1 Anesthesia and Critical Care, University Hospital of Derby, Derby, GBR; 2 Anesthesia and Pain Management, Shaukat Khanum Memorial Cancer Hospital and Research Centre, Lahore, PAK

**Keywords:** delphi process, informed consent, preconditions, simulated training, anaesthesia training

## Abstract

Introduction

Over the years, the process of obtaining informed consent has evolved and now places an emphasis on the concept that patients should play a major role in medical decision making. Failure to adequately involve patients in making decisions regarding their health can lead to medicolegal consequences.

Therefore, taking informed consent is a fundamental component of anaesthesia training. Simulation, for training, is an excellent tool that is being utilised widely in the training of medical professionals. The use of simulated training for teaching the process of informed consent is an innovative initiative that can provide improved results.

Material and methods

After approval from the institutional review board, a prospective clinical study was conducted at Shaukat Khanum Memorial Cancer Hospital and Research Centre, Lahore, from August 2019 to September 2020. Sixteen anaesthesia trainees were randomly selected for the study. The study was divided into pre-interventional, interventional and post interventional phases. For data collection, a predesigned checklist was used. Data collected was analysed using SPSS version 23 (IBM Inc., Armonk, New York). The McNemar test was deployed to assess the difference between the baseline assessment and post-simulated training assessment; p-value < 0.05 was taken to be significant.

Results

Of the 16 participants, the majority were males (n= 13). A positive impact was observed in terms of improvement of the outcome of the following study components i.e., description of benefits of the procedure (p=0.01), disclosure of associated minor risks (p=0.005), disclosure of major risks (p=0.01), discussion of alternatives (p=0.001), teach back (p=0.001), documentation of patients’ verbal agreement (p=0.01), and communication skills involving utilising the process of connecting, introduction, communication, permission, response, and exit (p = 0.01).

Conclusion

Simulated training had a positive impact in improving outcomes in the following study components: description of benefits of the procedure, disclosure of associated risks, discussion of alternatives, teach back, documentation of patients’ verbal agreement, and utilisation of the process of connecting, introduction, communication, permission, responding, and exiting.

## Introduction

The term “informed consent” was first formally introduced in the Court of Appeals, California, in 1957 [1]. Simply securing a signature on a given consent sheet cannot be deemed an appropriate substitute for the ongoing discussion between the patient and their physician. Patients possess the right to make their decisions about possible medical treatments made available to them. By navigating through the process of informed consent, the patients are allowed the necessary opportunity to avoid unwanted treatments; this also ensures that patients become responsible for their decisions. Taking proper informed consent also protects physicians against litigation. If a patient is treated without consent, this constitutes battery, while treatment without adequate consent constitutes negligence [2].

The documentation of consent is also important because it serves as proof that the process of informed consent did take place between concerned parties.

It is important to understand that the doctor-patient relationship has undergone a transition over time. The previously followed paternalistic approach has become redundant, and a patient is now an active participant in medical decision-making. There has been an evolutionary change in medical practice where the focus has shifted from the conventional to a modern approach towards informed consent. The conventional approach meant that doctors were expected to share information that they considered reasonable for the patient to be aware of; the modern practice makes it mandatory for them to share any material risks that a reasonable patient would like to know to be able to make an informed decision.

Certain historical case law and principles form the basis of evolution in the process of informed consent; the Bolam principle (1957), which laid down the basic principle regarding the standard of care being provided to patients in cases where there was alleged negligence, states that “a doctor is not guilty of negligence if he has acted in accordance with a practice accepted as proper by a responsible body of medical men skilled in that particular art” [3]. The Montgomery vs. Lanarkshire case (2015) makes it mandatory that doctors should share all information and options regarding treatment [4].

Informed consent is composed of three components: preconditions, information, and consent. “Preconditions” in this context means that a patient is competent as well as willing to grant consent for the proposed medical treatment. To exercise this right, the patient should possess the capacity to make such decisions and must be able to decide without being influenced by other individuals, which includes medical personnel [5].

The second component of informed consent is “information”. As per the World Health Organisation (WHO) resolution on the promotion of patients’ rights, patients have the right to receive full knowledge regarding the status of their health. This includes their current medical condition, information about the suggested medical procedure, any risks and benefits associated with the said procedure, and any possible alternatives to the planned procedure. The patient should also be educated about the possible effects on their health should they refuse to proceed with the proposed treatment [6]. The process of informed consent should also include a definite care plan including the physician’s advice, and it must be ensured that the patient has successfully comprehended the information shared with them [5].

Before proceeding with anaesthesia for any procedure, informed consent should involve the active participation of both the anaesthetist and the patient. During this conversation, it is the responsibility of the anaesthetist to disclose all information related to anaesthesia in a simplified manner that the patient or their guardian can understand and recall [7].

The process of taking informed consent can be difficult and time-consuming. Busy clinical schedules may make it challenging to allocate sufficient time required to obtain informed consent. One study estimated that an average time of 10.9 minutes was required to take appropriate informed consent [8]. The process of informed consent also requires maturity, patience, and self-awareness on the part of concerned physicians, so they do not take the liberty to abandon the seemingly tedious and time-consuming task of informed decision-making. Even though there is a consensus that the process of informed consent should be implemented in clinical practice, studies have shown that the theoretical ideal is rarely realised [9].

Taking informed consent is a fundamental skill expected to be mastered by anaesthesia residents during their training period. Informed consent forms a component of the anaesthesia patient care competency within the pre-anaesthetic evaluation, assessment, and preparation. Studies have shown that consent discussions are usually incomplete. These shortcomings of insufficient consent discussion are often responsible for increased litigation [10].

A study published in 2015 showed that simulated training of residents with standardised patients and faculty improved the ability of the residents to take informed consent [11]. Training of medical personnel is based on acquiring knowledge and skills by utilising real-life experiences. However, this technique is limited due to variations in types of supervision and feedback from actual patients. Having said this, the precise method to professionally train anaesthesia trainees in obtaining informed consent is yet to be developed. Simulated training in a well-designed and controlled environment is an innovative initiative that allows repeated training and accurate feedback [12,13].

## Materials and methods

This prospective clinical study was conducted at the Department of Anaesthesia, Shaukat Khanum Memorial Cancer Hospital, and Research Centre, Lahore, from July 2019 to September 2020, after approval from the Institutional Review Board.

Sixteen anaesthesia trainees, from the first to the fourth year of training, were randomly selected to participate in this study after their consent. A predesigned checklist, devised by Tanaka et al (Table 1), was used as an assessment tool. The study was conducted in three phases.

**Table 1:** Checklist used for assessment

1	Introduces self and the discussion topic
2	Describe the indications for the procedure
3	Describe the benefits of the procedure
4	Describe the procedure itself in clear, simple language
5	Pause for questions appropriately
6	Describe the minor risks of the procedure
7	Describe the risk of serious complications. Emphasise that these are rare
8	Describe alternatives to the procedure
9	Teach back: Ask the patient to repeat key items in the discussion
10	Have the patient verbally agree with the consent form
11	Utilised connect, introduce, communicate, ask permission, respond, exit

Phase One (Pre-intervention)

During this phase, a baseline assessment of the trainees' ability to obtain informed consent was carried out using simulated patients against the predesigned checklist.

Phase Two (Intervention)

This phase of the study comprised of a training session, which included a PowerPoint presentation and a pre-recorded video, aimed at covering important aspects of the consent-taking process. This was followed by a practice session using simulated patients. These practice sessions were video-recorded, which were then used to provide feedback to the trainees. The session was eventually followed by a post-simulation reassessment to determine the effectiveness of the training exercise.

Phase Three (Post Intervention)

**Figure 1:**
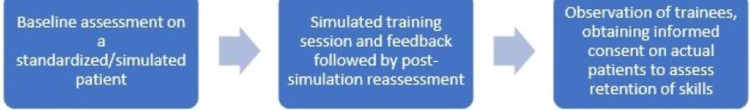
Phases of the study

After the intervention phase, a final assessment of the trainees was carried out. The trainees were asked to take informed consent from actual patients in a real clinical scenario. Their performance was assessed by employing the attached checklist (Figure 1). The purpose of this phase was to assess the degree of retention of skills by the trainees after having undergone simulated training.

Data was coded and analysed using SPSS version 23 (IBM Inc., Armonk, New York). With a confidence interval of 95% and a margin of error of 6.13%, the McNemar test was deployed to assess the difference between the baseline assessment and post-simulated training assessment.

The checklist comprising of 11 essential elements of the informed consent obtaining process used for assessing informed consent was adapted from a study done earlier at the University of Stanford in 2016 [10].

The checklist itself was made through the Modified Delphi Process. This process involves a systematic and interactive way of gaining opinions from a panel of independent experts to reach a consensus regarding a defined clinical problem [14].

## Results

**Figure 2:**
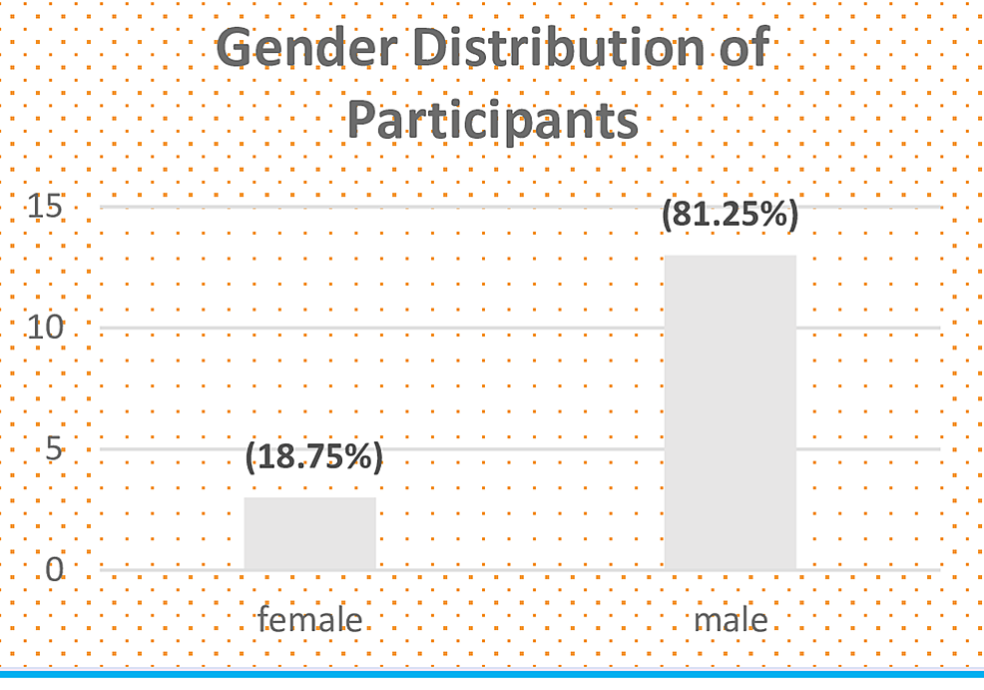
Gender distribution of participants

**Figure 3:**
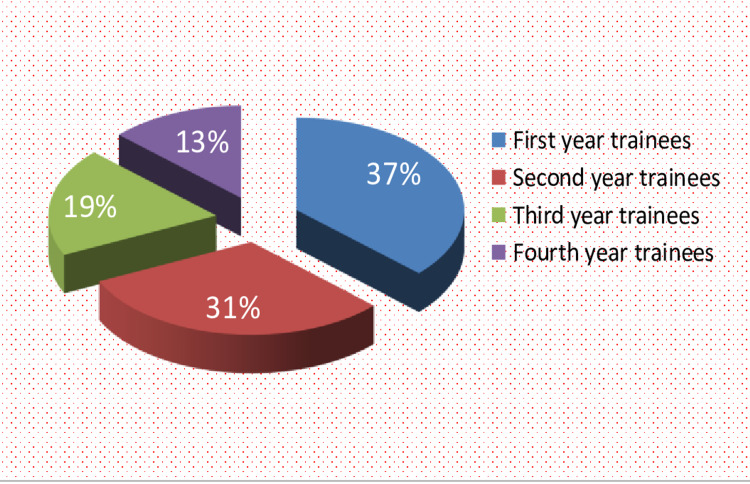
Participant demographics

A total of 16 residents took part in this study after random selection. The mean age of the residents was 29 ± 4 years. The majority of these were males, i.e. 13 (81.25%), and three (18.75%) were female (Figure 2). The study population comprised of trainees from a wide range of experience: six first-year residents, five second-year residents, three third-year residents, and finally, two residents in the fourth year of their training, with a mean of two years of experience in anaesthesia (Figure 3).

**Figure 4:**
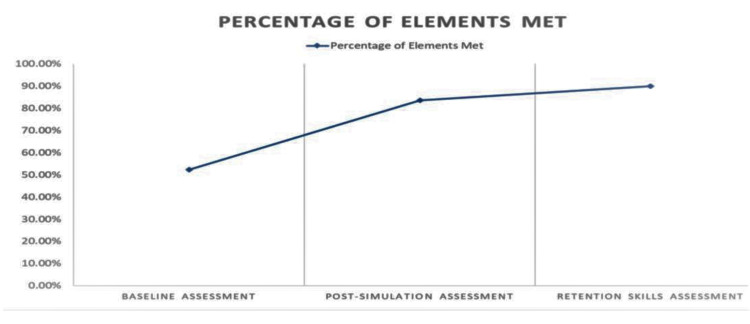
Elements met during the study

At baseline assessment, anaesthesia trainees were able to complete 52.2% of the required elements in the checklist. This percentage increased to 83.5% post-simulated training, which demonstrates the effectiveness of the training program. The final assessment conducted two to six weeks after the training showed that 89.9% (improvement of 37.7% from baseline) of the required elements were satisfactorily met by the trainees. This further proved that the training provided using simulation and feedback had long term benefits, as evidenced by the retention of the skills shown by the trainees (Figure 4).

**Figure 5:**
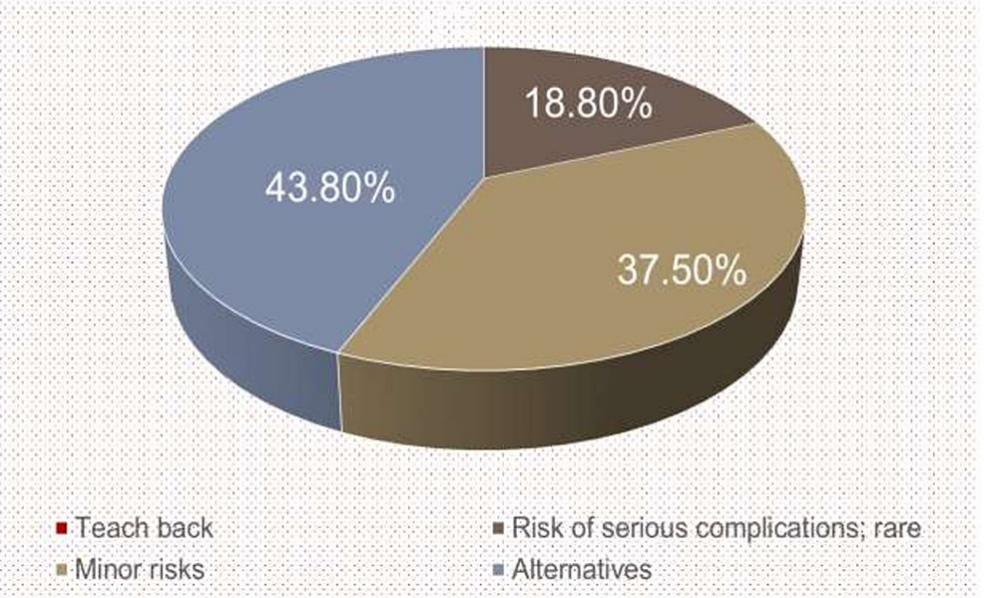
Poorly performed elements at baseline assessment

Certain elements that were most poorly met during baseline assessment were analysed; element nine, which is “teach back”, was most poorly covered, with all 16 residents failing to complete the requirement. Teach back is a method whereby a doctor asks the patient to repeat what they have understood from the conversation that has just taken place. This is meant to ensure that the message received is indeed the message intended. This was followed by element seven, which describes the risk of serious complications and emphasises that these are rare, at 18.8%. Element six describes the minor risks of the procedure at 37.5%. Element eight, which involves describing alternatives to the procedure, was completed by 43.8% of the residents (Figure 5).

A detailed comparison of elements met at baseline, post-simulation, and the final assessment was drawn, and it was observed that the percentage of all elements being met had increased from baseline to final assessment except for element one, which was met by all residents at baseline assessment.

**Figure 6:**
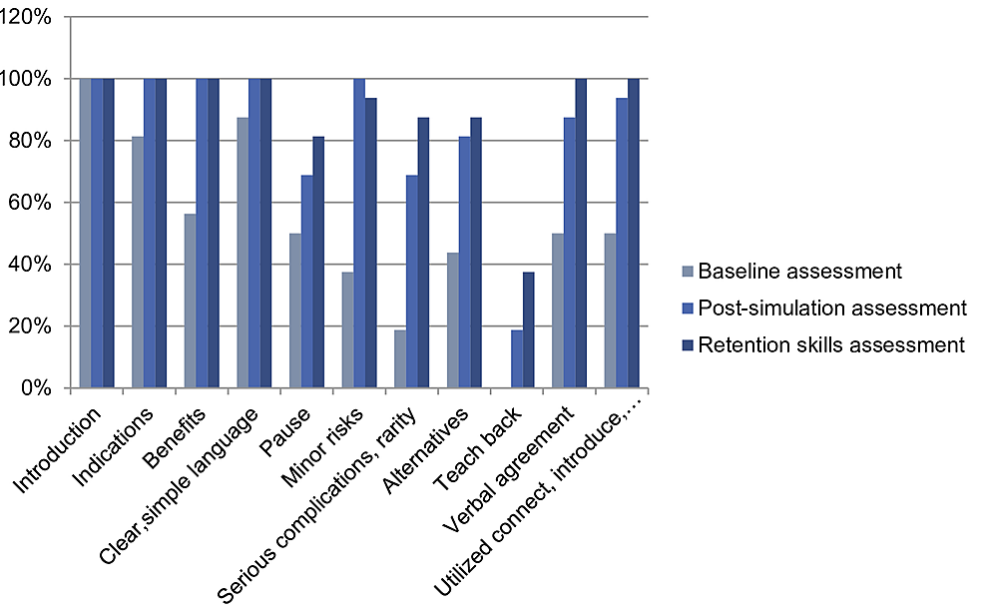
Comparison of elements met through the course of the study in the pre-intervention, intervention, and post-intervention phases

Teach back remained the most poorly met element at the final assessment; however, the percentage had gone up to 37.5% from a baseline of 0% (Figure 6).

A statistical analysis of the performance at baseline and final assessment using the McNemar test showed significant improvement in describing the benefits of the proposed procedure (p = 0.01), pausing for appropriate questions by the patient (p = 0.05), describing minor risks of the procedure (p = 0.005), describing the risk of serious complications and emphasising that these are rare (p = 0.01), describing alternatives to the procedure (p = 0.001), teach-back (p = 0.02), a verbal agreement to the proposed procedure (p = 0.01) and utilising the process of connecting, introduction, communication, permission, response and exit (p = 0.01) (Table 2).

**Table 2:** Statistical analysis of elements met at baseline and final assessment

Performed elements	Baseline assessment	Final assessment	p-value
Introduces self and the discussion topic	100%	100%	-
Describe the indications for the procedure	81.3%	100%	0.12
Describe the benefits of the procedure	56.3%	100%	0.01
Describe the procedure itself in clear, simple language	87.5%	100%	0.25
Pause for questions appropriately	50%	81.3%	0.05
Describe the minor risks of the procedure	37.5%	93.8%	0.005
Describe the risk of serious complications. Emphasise that these are rare	18.8%	87.5%	0.01
Describe alternatives to the procedure	43.8%	87.5%	0.001
Teach back: Ask the patient to repeat key items in the discussion	0%	37.5%	0.02
Have the patient verbally agree with the consent form	50%	100%	0.01
Utilised connect, introduce, communicate, ask permission, respond, exit	50%	100%	0.01

A comparison was drawn up between the trainees of 1st year through 4th year and it was noted that for all trainees, the percentage of elements met went up after the training session (Table 3).

**Table 3:** Comparison of elements met between trainees of each year.

Years of training	The average number of elements met at baseline assessment	The average number of elements met at final assessment
1	4	9.7
2	6.2	9.8
3	8.7	11
4	5.5	9

**Figure 7:**
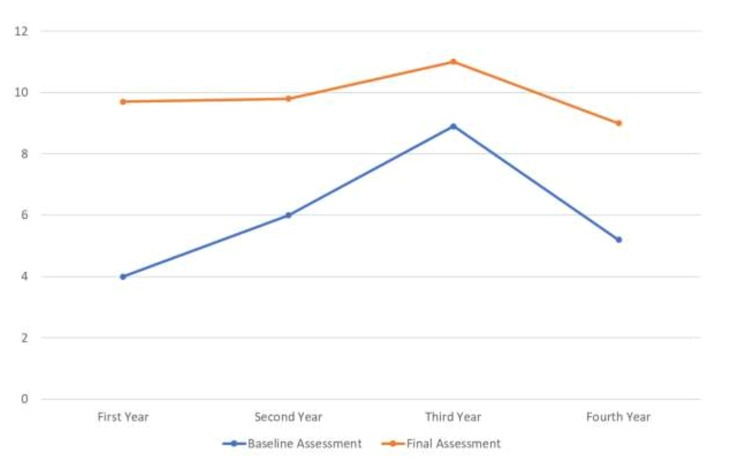
Comparative analysis of the trainees

It was also observed that with the increasing number of years of training the number of elements being met in an informed consent went up. This did not hold for the fourth-year residents (Figure 7).

## Discussion

For an anesthesiologist, the most important component of patient interaction is to obtain informed consent as it can have both ethical and legal implications [15]. Recently, The Association of Anaesthetists has also updated its recommendations for consent in anaesthesia in response to the changing ethical and legal background [16]. It is important to take informed consent in its true spirit which includes but is not limited to disclosure of relevant information, material risks, and alternatives while ensuring the patient fully comprehends the information delivered. It sounds simple, but in the real world, there are many obstacles in taking informed consent, for instance, a language barrier, limits on the time and physical space available for discussion, and the art of teaching this important communication skill to novice anaesthetists. Adequately explaining techniques and alternatives may take time in itself, in addition to the time required for patients to process, discuss and consider the options presented to them. Medical education has evolved from apprenticeships to dedicated teaching programmes and simulation is an effective way to teach procedural and non-technical skills within a safe environment, permitting expert feedback in real-time. It may also be used to demonstrate satisfactory achievement of competency before progressing to patient care [17]. This study aimed to assess whether simulated training can be incorporated into the teaching program of anaesthesia trainees working in busy operation theatres and whether any improvements in the consent-taking process could be achieved.

The data of this study shows that before simulated training, 50% of the participant residents thought that they could not obtain appropriate consent. However, the confidence in taking informed consent was raised to 100% after going through the training curriculum. The reflective result was also observed in other studies which showed the impact of simulation on improvement in confidence levels amongst study participants [14,15].

In most circumstances, patients are consented after being offered a single choice of anaesthetic with no alternative options. Moreover, the suggested anaesthetic is not explained to the full extent [18]. Similar results were observed in the current study, where during the pre-intervention phase, 56.3% of participants explained the benefits of the proposed anaesthetic, and only 43.8% discussed alternative options with the patients. However, marked improvement regarding these components of informed consent, i.e., explanation of benefits (p=0.01) and disclosure of alternates (p=0.001), was observed in the post-intervention phase.

“Teach back”, which is asking the patient to describe what has just been discussed in their own words, was the most poorly performed element in baseline assessment amongst the participants of the current study (0 out of 16 participants at pre-assessment). This was also the case in the study performed by Tanaka et al. [10]. However, post-simulation significant improvement (p = 0.02) was observed at the final assessment.

Disclosure of information about major and minor risks associated with each anaesthetic and procedure is an important element of any informed consent. This study assessed the ability of the participants to provide patients with this information. Significant improvement in providing information about minor risks was observed post-simulation (37.5% to 93.8%, p-value: 0.005). Discussion about major risks, which is often overlooked by physicians due to fear of causing distress, showed an improvement from 18.8% on the initial assessment to 87.5 % at the final assessment with a p-value of 0.01. A study conducted by Jawaid et al., comprising of 307 patients, also highlighted this neglected component of informed consent, i.e., only 4.9 % of the patients were made aware of the complications and risks associated with anaesthesia and procedures [19].

Documentation of patients’ verbal agreement to the suggested anaesthetic is a key requirement of informed consent as per the guidelines set forth by The Association of Anaesthetists of Great Britain and Ireland (AAGBI) [16]. This aspect of the informed consent showed an improvement of 50% (p=0.01) pre and post-simulated training in the study under discussion.

The use of simulators to train anaesthetists has been in use since 1969 when Denson and Abrahamson invented the first simulator, SIM 1. Their use is widespread in simulating emergencies within a theatre environment. This has led to significant improvement in management outcomes in various disciplines of medicine, including anaesthesia [20-23]. The process of simulated training on performance improvement was also demonstrated in this study, where an improvement from 52.2% to 83.5% occurred in the overall consent-taking process after simulated training. We, therefore, suggest that similar studies involving a larger population of anaesthesia trainees should be conducted. This will be a daunting task; however, it can be taken up by the training bodies, which can make such simulation courses an essential component of core competencies required to progress as anaesthesia trainees.

## Conclusions

Simulation is well established as an important educational tool in anaesthesia and can be used effectively to teach non-technical skills like taking informed consent. In our study, simulated training had a positive impact in improving outcomes in the following study components: description of benefits of the procedure, disclosure of associated risks, discussion of alternatives, teach back, and documentation of patients’ verbal agreement.

## References

[1] Chan Y, Irish JC, Wood SJ, Rotstein LE, Brown DH, Gullane PJ, Lockwood GA (2002). Patient education and informed consent in head and neck surgery. Arch Otolaryngol Head Neck Surg.

[2] Etchells E, Sharpe G, Walsh P, Williams JR, Singer PA (1996). Bioethics for clinicians: 1. Consent. CMAJ.

[3] LawTeacher. Bolam v Friern Hospital Management Committee [Internet (2013). LawTeacher. Bolam v Friern hospital management committee. November.

[4] Mallon Mallon, C. (2019, June 23). Montgomery v Lanarkshire Health Board (2019). Montgomery v Lanarkshire health board. https://chrismallonlawtutor.com/medical-law/montgomery-v-lanarkshire-health-board/.

[5] Leclercq WK, Keulers BJ, Scheltinga MR (2010). A review of surgical informed consent: past, present, and future. A quest to help patients make better decisions. World journal of surgery.

[6] Universiteit van Amsterdam Health Law Section (1995). Promotion of the rights of patients in Europe: proceedings of a WHO consultation. World J Surg.

[7] Rai E, Chen RY, Noi CS, Hee HI (2019). Evaluation of anesthesia informed consent in pediatric practice - an observation cohort study. J Anaesthesiol Clin Pharmacol.

[8] Fink AS, Prochazka AV, Henderson WG (2010). Enhancement of surgical informed consent by addition of repeat back: a multicenter, randomized controlled clinical trial. Ann Surg.

[9] Hall DE, Prochazka AV, Fink AS (2012). Informed consent for clinical treatment. CMAJ.

[10] Tanaka P, Park L, Tanaka M, Udani DA, Macario A (2016). Development and testing of a curriculum for teaching informed consent for spinal anesthesia to anesthesiology residents. J Pain Relief.

[11] Thompson BM, Sparks RA, Seavey J (2015). Informed consent training improves surgery resident performance in simulated encounters with standardized patients. Am J Surg.

[12] Ericsson KA (2015). Acquisition and maintenance of medical expertise: a perspective from the expert-performance approach with deliberate practice. Acad Med.

[13] Macario A (2014). Can physician performance be assessed via simulation?. Anesthesiology.

[14] Woodcock T, Adeleke Y, Goeschel C, Pronovost P, Dixon-Woods M (2020). A modified Delphi study to identify the features of high quality measurement plans for healthcare improvement projects. BMC Med Res Methodol.

[15] Potgieter HE (2020). Authenticity of informed consent in anaesthesia: ethical reflection on the dilemma of informed consent in anaesthesia. http://scholar.sun.ac.za/handle/10019.1/108070.

[16] Yentis SM, Hartle AJ, Barker IR (2017). AAGBI: Consent for anaesthesia 2017: Association of Anaesthetists of Great Britain and Ireland. Anaesthesia.

[17] Maran NJ, Glavin RJ (2003). Low- to high-fidelity simulation - a continuum of medical education?. Med Educ.

[18] Braun AR, Skene L, Merry AF (2010). Informed consent for anaesthesia in Australia and New Zealand. Anaesth Intensive Care.

[19] Jawaid M, Farhan M, Masood Z, Husnain S (2012). Preoperative informed consent: is it truly informed?. Iran J Public Health.

[20] Yunoki K, Sakai T (2018). The role of simulation training in anesthesiology resident education. J Anesth.

[21] Chopra V, Gesink BJ, de Jong J, Bovill JG, Spierdijk J, Brand R (1994). Does training on an anaesthesia simulator lead to improvement in performance?. Br J Anaesth.

[22] Meska MH, Mazzo A, Jorge BM, Souza-Junior VD, Negri EC, Chayamiti EM (2016). Urinary retention: implications of low-fidelity simulation training on the self-confidence of nurses. Rev Esc Enferm USP.

[23] İsmailoğlu EG, Orkun N, Eşer İ, Zaybak A (2020). Comparison of the effectiveness of the virtual simulator and video-assisted teaching on intravenous catheter insertion skills and self-confidence: a quasi-experimental study. Nurse Educ Today.

